# Antenatal care in The Gambia: Missed opportunity for information, education and communication

**DOI:** 10.1186/1471-2393-8-9

**Published:** 2008-03-07

**Authors:** Samuel E Anya, Abba Hydara, Lamin ES Jaiteh

**Affiliations:** 1 Reproductive Health Research Unit, School of Medicine and Allied Health Sciences, University of The Gambia, MDI Road, Kanifing, The Gambia; 2 School of Medicine and Allied Health Sciences, University of The Gambia, MDI Road, Kanifing, The Gambia

## Abstract

**Background:**

Antenatal care is widely established and provides an opportunity to inform and educate pregnant women about pregnancy, childbirth and care of the newborn. It is expected that this would assist the women in making choices that would contribute to good pregnancy outcome. We examined the provision of information and education in antenatal clinics from the perspective of pregnant women attending these clinics.

**Methods:**

A cross sectional survey of 457 pregnant women attending six urban and six rural antenatal clinics in the largest health division in The Gambia was undertaken. The women were interviewed using modified antenatal client exit interview and antenatal record review questionnaires from the WHO Safe Motherhood Needs Assessment kit. Differences between women attending urban and rural clinics were assessed using the Chi-square test. Relative risks with 95% confidence intervals are presented.

**Results:**

Ninety percent of those interviewed had attended the antenatal clinic more than once and 52% four or more times. Most pregnant women (70.5%) said they spent 3 minutes or less with the antenatal care provider. About 35% recalled they were informed or educated on diet and nutrition, 30.4% on care of the baby, 23.6% on family planning, 22.8% on place of birth and 19.3% on what to do if there was a complication.

About 25% of pregnant women said they were given information about the progress of their pregnancy after consultation and only 12.8% asked their provider any question. Awareness of danger signs was low. The proportions of women that recognised signs of danger were 28.9% for anaemia, 24.6% for hypertension, 14.8% for haemorrhage, 12.9% for fever and 5% for puerperal sepsis. Prolonged labour was not recognised as a danger sign. Women attending rural antenatal clinics were 1.6 times more likely to recognise signs of anaemia and hypertension as indicative of danger compared to women attending urban antenatal clinics.

**Conclusion:**

Information, education and communication during antenatal care in the largest health division are poor. Pregnant women are ill-equipped to make appropriate choices especially when they are in danger. This contributes to the persistence of high maternal mortality ratios in the country.

## Background

Antenatal care provides an opportunity to inform and educate pregnant women on a variety of issues related to pregnancy, birth and parenthood. The aim of this exercise is to equip them to make appropriate choices that will contribute to optimum pregnancy outcome and care of the newborn [1]. This concept has made antenatal education programmes a standard component of antenatal care worldwide [2].

There has been controversy about the impact of antenatal education on pregnancy outcome [3]. However, a recent synthesis of experience with information, education and communication (IEC) makes the point that it works [4]. In other words, an appropriate strategy of IEC leads to or reinforces desirable attitudes and behaviour.

Educated women have better pregnancy outcome compared with uneducated women [5,6]. This may be partly because they are better informed and make better choices. Literacy among women in many developing countries is low and there are socio-cultural beliefs and practices with adverse effects on pregnancy and birth even among educated women [7]. The "Three Phases of Delay Model" highlights the importance of IEC in the prevention of maternal death by describing the sequence of events from late recognition of danger signs to maternal death [8]. Therefore, an appropriate programme of health literacy or behaviour change communication is highly desirable.

In The Gambia, over 90% of pregnant women visit the antenatal clinic at least once and 70% attend four or more times [9]. This high antenatal coverage and relatively high frequency of visits provides an excellent opportunity for information, education and communication. The principal guiding documents for the delivery of maternity services in The Gambia show that the provision of information, education and communication is a major component of antenatal care [10-12]. The IEC strategy was first articulated in the early 1990s but has undergone revisions in line with emerging needs. The strategy is to inform and educate pregnant women on a variety of topics including nutrition, malaria, STIs/HIV/AIDS, danger signs of pregnancy and delivery, and care of the newborn. Furthermore, antenatal care providers are expected to engage in dialogue with pregnant women to develop appropriate and individualized delivery plans based on the concept of birth preparedness and complication readiness [12]. This strategy should be implemented routinely in all antenatal clinics.

Despite the foregoing, a recent qualitative study of maternal deaths suggested that low levels of awareness of danger signs of pregnancy and delivery contribute to continuing high maternal mortality ratios in the country [13].

To further explore this issue, we undertook a study to: (i) determine the experiences of pregnant women with antenatal IEC; (ii) determine their levels of awareness of danger signs during pregnancy and delivery; and (iii) compare experiences and levels of awareness between pregnant women attending urban and those attending rural antenatal clinics in the largest health division in The Gambia. We sought to compare urban and rural antenatal clinics because maternal mortality ratios in rural areas were two times higher than in urban areas in the most recent national maternal mortality survey [9]. This data will be useful for reinforcing or modifying behaviour change communication for pregnant women as part of an overall strategy for achieving the country's Millennium Development Goal for Maternal Health.

## Methods

The Western Health Division, one of six health divisions, comprises three local government councils – Banjul, Kanifing and Brikama. It covers 17.3% of the geographical area of the country but accommodates 55% of the national population [14].

Most antenatal care is provided through government facilities (80–85%) and Non-Governmental Organizations (15–20%). Less than 3% of pregnant women receive antenatal care at private facilities [9]. Registration at a public sector antenatal clinic depends on place of residence. There are six base antenatal clinics in urban areas and five base antenatal clinics in rural areas in Western Health Division. Urban or rural status was assigned based on definitions laid down by the Central Statistics Department of The Gambia [14]. Antenatal clinics open on week days from 8.00 a.m. to 2.00 p.m. and are led by trained midwives. Pregnant women are usually given monthly appointments until 28 weeks gestation, two-weekly appointments until 36 weeks and then weekly appointments until birth. The programme of activities for each clinic session includes a health talk; assessment of pregnant women through history taking, examination and laboratory tests (as indicated) and; provision of tetanus toxoid immunizations and iron/folate supplementation. Health talks are intended to cover nutrition, malaria, STIs/HIV/AIDS, danger signs of pregnancy and delivery, family planning, breastfeeding and care of the newborn. Other management and/or referral take place as indicated. In addition to the above routine, services to prevent parent to child transmission of HIV were offered at two of the facilities as a pilot programme. Staff at these facilities received additional training in order to provide the service. At some base clinics, staff "trekked" to outreach posts to conduct antenatal clinics for parts of their catchment population.

We adapted the Antenatal Client Exit Interview and Antenatal Record Review questionnaires in the Safe Motherhood Needs Assessment (SMNA) Kit [15]. The questionnaires were translated into two local languages (Mandinka and Wolof) and back-translated to ensure consistency.

To achieve a 95% confidence level with a 10% confidence interval, we required a sample size of 192 pregnant women based on an estimated number of 30,000 maternities per year in Western Health Division [15]. Since we intended to compare pregnant women at rural and urban clinics, we determined the sample size for rural and urban women separately giving a desired sample size of 384 (192 rural and 192 urban). We aimed to interview 50 pregnant women at large (busy) antenatal clinics and 25 pregnant women at smaller centres for proportional representation. If there were more women than required at the clinic, systematic random sampling was done. If there were fewer women than required, all were selected and a second visit was made to the clinic to complete the required number.

All pregnant women attending the antenatal clinic when the interviewers were present were eligible to participate. Pregnant women who had emergency conditions were excluded. Individual women were approached, given information regarding the purpose of the study, invited to participate, assured of confidentiality and reassured that opting out would not compromise the care they would receive. Out of 459 women who were approached, 457 participated in the survey. The two who opted out did not give any reasons. The high response rate is not surprising because surveys are common among women of reproductive age in The Gambia. AH and LESJ interviewed the selected women individually and in private at the end of their antenatal clinic consultation. Interviews began in the morning and continued into the afternoon. The study took place in November and December 2004.

The differences between women attending urban and rural clinics were assessed using the Chi-square test. Relative risks with 95% confidence intervals are presented.

The study was approved by the Department of State for Health and Social Welfare, Banjul. The pregnant women who participated gave individual informed consent.

## Results

### Characteristics of antenatal clients

We analyzed questionnaires from 457 antenatal clients at six urban base clinics, four rural base clinics and two rural outreach antenatal clinics. The characteristics of the respondents are presented in Table 1. Most women attended the antenatal clinic for the first time when pregnancy was advanced. The proportion of women who had their first visit at 20 weeks or less was 25.6% (117/457). Only 10% of women were attending the antenatal clinic for the first time on the day of the interview.

**Table 1:** Characteristics of antenatal clients

Characteristic	Percentage (%)
**Age distribution (years)**	N = 447
< 20	17.7
20–24	33.6
25–29	29.1
30–34	12.3
35+	7.4

**Gravidity**	N = 456
1	23.9
2	21.3
3	16.0
4	12.7
5	10.5
6+	15.6

**Gestational age at first visit**	N = 457
First trimester (0–13 weeks)	8.1
Second trimester (14–27 weeks)	62.8
Third trimester (28+ weeks)	29.1

**Number of visits at interview**	N = 457
1	10
2–3	38
4+	52

### Provision of information, education and communication

Table 2 displays the proportion of antenatal clients who reported that they had received information, education or counseling (IEC) on the selected topics at any of their antenatal visits. According to the respondents, diet and nutrition was the topic most likely to have been discussed. However, only about one-third of the women reportedly benefited from this. Less than 25% of the pregnant women recalled having discussed child spacing or family planning with their antenatal provider. Provision of IEC on what to do in an emergency was recalled by even fewer women.

**Table 2:** Women provided with IEC on selected topics at any or all antenatal visits.

**Topic**	N = 457	
	
	**n**	**%**
Diet and nutrition	162	35.4
How to get to health facility if there were an emergency	142	31.1
Benefit of birth in a health facility	140	30.6
Care of your baby	139	30.4
STIs, HIV/AIDS	137	30.0^‡^
Child spacing/family planning	108	23.6
Place of birth	104	22.8
What to do if there is a problem such as bleeding or convulsions/fits	88	19.3

^‡^86/101 (85.1%) at the two health facilities where prevention of parent-to-child transmission of HIV (PPTCT) was being piloted compared to 51/356 (14.3%) at other health facilities where there was no PPTCT programme.

Among women with one or more prior deliveries (346), 13% (45) had a previous caesarean section, stillbirth or convulsion. However, only 28.9% (13/45) of this high-risk group said they were advised to give birth at a health facility in the index pregnancy.

### Provider-client interaction

Communication between antenatal care provider and antenatal client was poor. One quarter of pregnant women said they were told about the progress of their pregnancy following consultation on the day of the interview (Table 3). An even smaller proportion of clients (12.8%) asked their provider any question.

**Table 3:** Communication between health workers and pregnant women following consultation on day of interview.

	N = 452	
	
**The antenatal client reported**	**n**	**%**
being told progress of pregnancy	115	25.4
being asked the antenatal care provider questions	58	12.8
having understood answers given by provider	55*	94.8
being asked to return for another visit	312**	69.5

*N = 58 (those that asked questions)

**N = 449

Only 2.5% (11/438) said they spent 10 or more minutes with the provider while 70.5% of women (309/438) said they spent 3 minutes or less with their antenatal care provider. About 64% (287/450) felt they were attended to with adequate privacy.

### Awareness of danger signs in pregnancy

Table 4 shows that signs related to anaemia and hypertensive disorders in pregnancy were the commonest listed by respondents but only by relatively small proportions of them. Only 14.8% of pregnant women listed heavy bleeding as a danger sign. However 30.8% (39/127) of women in their fourth or more pregnancy did so compared to 10.3% (23/224) of women who had fewer pregnancies. This difference was statistically significant (Relative risk = 2.5; 95% confidence interval = 1.6–4.1; p = 0.0001). Apart from this multi-gravidity and higher number of visits were not significantly associated with higher levels of awareness of danger signs.

**Table 4:** Proportion of pregnant women that were aware of danger signs in pregnancy.

**Pregnancy-related danger signs**	N = 418	
	
	**n**	**%**
Anaemia/pallor/fatigue/breathlessness	121	28.9
Hypertension/headache/swelling/fits	103	24.6
Haemorrhage/heavy bleeding	62	14.8
Sepsis/foul smelling discharge/postpartum abdominal pain	21	5.0
Cessation of fetal movement/baby does not move	13	3.1
Light bleeding/spotting	9	2.2
Abnormal lie/position of fetus	3	0.7
Multiple pregnancy/large abdomen	0	0.0
Difficult/obstructed/prolonged labour/"sun set two times"	0	0.0
Previous bad obstetric history/abdominal scars/previous stillbirth	0	0.0

Under "other signs", abdominal pain was the commonest condition considered indicative of danger (Table 5).

**Table 5:** Other signs considered indicative of danger by pregnant women

**Other signs considered suggestive of danger**	N = 418	
	**n**	**%**
Abdominal pain	181	43.3
Fever/malaria	54	12.9
Backache/waist pain/pain	31	7.4
Illness (unspecified)	25	6.0
Vomiting	12	2.9
Chest pain	11	2.6
Early labour	11	2.6
Drainage of liquor	9	2.2
Labour	8	1.9
Poor appetite	4	1.0
Cough	3	0.7
Asthma/diabetes	1	0.2

### Differences between women attending rural and urban antenatal clinics

Pregnant women who received antenatal care in rural areas (202) and those that received antenatal care in urban areas (255) were similar in most respects. Out of 13 variables tested, the only variables with statistically significant differences related to privacy and awareness of two danger signs in pregnancy with pregnant women attending rural antenatal clinics at an advantage (Table 6).

**Table 6:** Comparison of experiences and awareness of danger signs between pregnant women attending rural and urban antenatal clinics.

**Characteristic**	**Rural pregnant women n/N (%)**	**Urban pregnant women n/N (%)**	**Relative Risk Ratio Rural/Urban (95% confidence interval)**	**p value**
Adequate privacy	146/202 (72.3)	141/248 (56.9)	1.3 (1.1 – 1.5)	0.001
*Awareness of danger signs*				
Anaemia/pallor/fatigue/breathlessness/dizziness	69/187 (36.9)	52/231 (22.5)	1.6 (1.2 – 2.2)	0.002
Hypertension/headache/swelling/fits	58/187 (31)	45/231 (19.5)	1.6 (1.1 – 2.2)	0.009

*Antenatal client*...				
Said she spent 3 minutes or less with provider	136/192 (70.8)	173/246 (70.3)	1.0 (0.9–1.1)	0.84
Was told progress of pregnancy	51/202 (25.2)	64/250 (25.6)	1.0 (0.7–1.4)	0.98
Asked the antenatal care provider questions	32/202 (15.8)	26/250 (10.4)	1.5 (0.9–2.5)	0.11
Understood answers given by provider	28/33 (84.8)	27/32 (84.4)	1.0 (0.8–1.2)	0.77
Was asked to return for another visit	140/200 (70.0)	172/249 (69.1)	1.0 (0.9–1.2)	0.91
Had complications in previous pregnancy and was advised to deliver at a health facility in current pregnancy	7/25 (28.0)	6/20 (30.0)	0.9 (0.4–2.3)	0.85
*Awareness of danger signs*				
Haemorrhage/heavy bleeding	24/187 (12.8)	38/231 (16.5)	0.8 (0.5–1.3)	0.37
Sepsis/foul smelling discharge/postpartum abdominal pain	5/187 (2.7)	16/231 (6.9)	0.4 (0.1–1.0)	0.08
Cessation of fetal movement/baby does not move	6/187 (3.2)	7/231 (3.0)	1.1 (0.4–3.1)	0.86
Light bleeding/spotting	2/187 (1.1)	7/231 (3.0)	0.4 (0.1–1.7)	0.3

## Discussion

The results of this study show that although a large proportion of women attending antenatal clinics did so repeatedly, they were not benefiting from effective information, education and communication which together form one of the primary purposes of antenatal care. Ninety percent of those interviewed had attended the antenatal clinic more than once and 52% four or more times. However, most of them (70.5%) said they spent 3 minutes or less with the antenatal care provider and less than 40% could recall being informed or educated about important subjects such as diet and nutrition, care of the baby, family planning, place of birth. An even smaller proportion (19.3%) could recall being educated about what to do if there was a complication.

One of the lessons learned from the Safe Motherhood Initiative is that community involvement is a key requirement for sustainable reduction of maternal mortality [16]. However, individual women, families and communities need information and education to be empowered to contribute positively to making pregnancy safer. Therefore, the large proportion of pregnant women in this study who reported that they were not informed or educated about important issues is of concern.

We observed that pregnant women were least likely to recall having received the IEC that related to the recognition of danger and response to pregnancy-related problems. In a study of women 6–12 months after giving birth in another health division, Walraven et al found even lower levels of antenatal education on danger signs and complication readiness [17]. This low level of awareness of danger signs contributes to a failure to obtain adequate care in time and hence, maternal death in The Gambia [13]. There was even less awareness about danger signs for the unborn child.

Information, education and communication require time. The new antenatal care model recommends 30–40 minutes for the first visit and 20 minutes for subsequent visits to carry out all activities including individual IEC [18]. However, most women said they spent 3 minutes or less with their provider. Communicating effectively under this circumstance would be an enormous challenge and could explain the poor provider-client interaction. It was not surprising that in this "rushed" scenario very few women asked questions. On the other hand, it is encouraging that 95% of women understood the answers given to the questions they asked. Direct observation in Nepal revealed that an average of only one minute was spent on counseling [19]. Little attention was also paid to danger signs and complication readiness with communication flowing mostly in one direction from health worker to pregnant woman. Although, the health system in Nepal is probably different from ours, it suggests that the challenge to provide adequate antenatal IEC is not unique to our setting. On the other hand, the new antenatal care model also recommends fewer routine antenatal visits with the expectation that this would give providers more time to spend communicating effectively with clients.

Staff shortages are a major constraint in the delivery of health services in The Gambia but midwives are particularly affected by excessive workloads [20]. Since each midwife must attend to a relatively large number of women in a defined period, the provision of IEC may be given less priority. Even where there are adequate numbers, staff training, attitudes, supervision and incentives would be important determinants of the quality of the services that are delivered. We did not assess these issues but believe that they would be important for planning and organization of services.

There are efforts to improve the human resource situation but in the short term, an effective IEC strategy is needed. Traditionally, IEC has been provided at the antenatal clinic level. In view of the human resource constraints and the need to reach a wider audience, we propose a strategy that provides IEC through the mass media – addressing various issues and encouraging women to ask questions at the antenatal clinic. This has several advantages. A uniform message will be disseminated, it will reach non-pregnant women who will be better informed and able to make better choices early on when they become pregnant (such as early attendance which is particularly important for effective intermittent preventive treatment of malaria). These messages will also reach men (who are influential in maternal health) to encourage positive participation as partners in making pregnancy safer. Cultural perceptions at family and community levels influence how the woman, family and community respond to pregnancy-related complications [15]. By using the mass media, family members and the community as a whole would also be reached with messages that encourage positive attitudes and participation. This is more consistent with intentions of the National Reproductive Health Policy [11].

Ensuring messages reach the target audience is a concern. However, there is recent positive experience in the country with the use of radio for IEC for increasing awareness and knowledge of HIV/AIDS [21]. In Guatemala, clinic and population-based surveys demonstrated that pregnancy-related radio messages increased awareness of danger signs in pregnancy and were an important complement to clinic-based education [22].

The nationwide Maternal Mortality Survey showed that women in rural areas were at increased risk of maternal death [9]. However, the reported provision of IEC was similar in both urban and rural antenatal clinics and where differences occurred, women attending rural antenatal clinics performed better. Thus, the higher risk of death among rural women was not because they were less likely to recognize danger compared to women in urban areas. A possible explanation is that delivery services are less readily available to rural women compared to urban women. In rural communities, access to antenatal services is enhanced through outreach ("trekking") clinics. Health workers from designated base clinics travel to the communities on specific days to provide antenatal services and return to their base. For delivery, women in these rural communities have to travel to the base clinic. Thus, the distance barrier has been overcome for antenatal services but not for delivery services. This is a critical issue since most complications that lead to maternal death occur around the time of delivery.

## Conclusion

Pregnant women who do not have adequate and appropriate information about pregnancy and childbirth would be ill-equipped to make choices that will contribute to their own well-being. Delivering carefully developed messages through the mass media, especially the radio is an attractive and feasible strategy that has proven successful with HIV/AIDS in the country. Another vital part of the strategy would be to clearly identify the barriers to individual counseling at the clinic level and institute appropriate interventions to ensure that the peculiar circumstance of each pregnant woman is dealt with.

On the other hand, pregnant women would be unable to make optimal use of the information they have been provided if services are not readily available. Therefore, improving access to services that they have been advised to make use of is vital. In our context, this would mean improving the access to skilled attendance at delivery particularly for rural women.

We believe these steps would contribute to meeting the United Nations millennium development goals for maternal and newborn heath as well as for gender equality and empowerment of women in The Gambia.

## Competing interests

The author(s) declare that they have no competing interests.

## Authors' contributions

SEA conceived the study, contributed to the design, analyzed the results and wrote the first draft. AH and LESJ contributed to the concept and design of the study, collected the data and contributed to the drafting the manuscript. All authors read and approved the final manuscript.

## Pre-publication history

The pre-publication history for this paper can be accessed here:


